# Impact of Educational Interventions on the Quality of Life of Patients With Stage 4-5 Chronic Kidney Disease

**DOI:** 10.7759/cureus.81839

**Published:** 2025-04-07

**Authors:** Marianna A Eleftheroudi, Efstathios Mitsopoulos, Dorothea Papadopoulou, Gerasimos Bamichas, Apostolos Kyrgialanis, Fani Papoulidou, Elias Thodis, Maria Samakouri, Stylianos Panagoutsos, Ploumis Passadakis

**Affiliations:** 1 Nephrology, Papageorgiou General Hospital, Thessaloniki, GRC; 2 Nephrology, Papanikolaou General Hospital, Thessaloniki, GRC; 3 Nephrology, Xanthi General Hospital, Xanthi, GRC; 4 Nephrology, Kavala General Hospital, Kavala, GRC; 5 Nephrology, University Hospital of Alexandroupolis, Medical School, Democritus University of Thrace, Alexandroupolis, GRC; 6 Psychiatry, University Hospital of Alexandroupolis, Medical School, Democritus University of Thrace, Alexandroupolis, GRC

**Keywords:** anxiety, chronic kidney disease (ckd), depression, educational intervention, quality of life (qol)

## Abstract

Background and objectives

Chronic kidney disease (CKD) and its management can adversely affect patients' quality of life and mental health. Many patients are often inadequately informed about their condition and the available treatment options. A considerable number of people with advanced kidney disease have insufficient knowledge about their condition and the available treatment options. This study aims to investigate the impact of an educational intervention on quality of life, psychological resilience, anxiety, and depression. This study specifically aims to evaluate the effectiveness of this intervention on variables such as quality of life, psychological resilience, anxiety, and depression.

Methods

A prospective randomized study was conducted on patients with CKD stage 4-5 to evaluate the effectiveness of an educational intervention in improving the aforementioned parameters. Furthermore, the correlation between these outcomes and the level of satisfaction with the intervention was analyzed. Participants were randomized (3:1) into two groups: an educational intervention group (33 patients) and a control group (12 patients). In the intervention group, we performed educational presentations accompanied by a relevant video. Participants completed the questionnaires both before and after the intervention. Participants in the control group were only asked to complete the questionnaires again after the same time period as the intervention group.

Results

Higher satisfaction with the intervention was significantly associated with a reduction in anxiety (r = -0.38, p = 0.03) and depression scores (r = -0.44, p = 0.01), along with a notable improvement in social functioning (r = 0.40, p = 0.02). Furthermore, a marginally significant positive correlation was observed between the satisfaction scale and psychological resilience (r = 0.32, p = 0.07).

Conclusion

The level of satisfaction with the intervention is a critical factor in determining its effectiveness. Tailoring the intervention to address the unique needs of each patient may significantly enhance its impact.

## Introduction

Chronic kidney disease (CKD) is one of the most prevalent chronic conditions, affecting approximately 10% of the global population, according to recent estimates by the International Society of Nephrology (2024). It is well-established that both the disease itself and its treatment negatively impact patients' quality of life and significantly affect their mental health [1]. More specifically, kidney replacement therapies appear to influence not only the physical health and functionality of patients but also their interpersonal, social, and professional lives as a whole [2].

Several studies suggest that the more informed patients are about their disease and its treatment, the better their disease outcomes tend to be [3]. However, at least 60% of patients with CKD, regardless of the stage of the disease, lack basic knowledge about the condition, therapeutic options, and their mechanisms [4-6]. Only 9% of patients demonstrate satisfactory awareness of these aspects [7]. To address this knowledge gap, various educational programs have been implemented, proving effective in enhancing patient awareness of their condition [8]. Notably, a recent review of 120 studies highlighted the different forms of therapeutic educational interventions for CKD patients [9].

Therapeutic patient education refers to an educational intervention aimed at improving clinical parameters such as adherence to treatment guidelines, self-care techniques, self-efficacy, and overall quality of life. These interventions are conducted by trained healthcare professionals [10]. Such programs empower patients to self-manage chronic conditions with the support of caregivers or family members by providing essential information about the disease, thereby enhancing their ability to participate in shared decision-making [10]. It is important to note that these interventions are not intended as a substitute for physical treatments or structured psychotherapeutic interventions such as cognitive behavioral therapy (CBT). Instead, they are considered a form of supportive complementary therapy [11].

Various organizations, such as the World Health Organization (2023b), have developed protocols for therapeutic patient education. Additionally, the integration of such interventions into routine nephrology practice has been proposed [12]. Educating patients about their disease has been shown to directly influence their psychological well-being, quality of life, adherence to treatment, and even disease progression [13-19].

Considering the significant burden CKD places on patients' quality of life, the design and implementation of an educational intervention aimed at providing holistic support were deemed necessary. This study specifically aims to evaluate the effectiveness of this intervention on variables such as quality of life, psychological resilience, anxiety, and depression.

## Materials and methods

Study overview

A prospective randomized study was conducted on patients with stage 4-5 CKD. Participants were divided into two groups: the educational intervention group and the control group. The effectiveness of the educational intervention was evaluated using validated self-report questionnaires to assess changes in psychological resilience, depression, anxiety, and quality of life among patients. The effectiveness of the educational intervention was then evaluated in terms of increasing levels of psychological resilience, reducing depression and anxiety levels, and improving the patients’ quality of life. Finally, the correlation between these outcomes and the level of satisfaction with the intervention was examined.

Ethical considerations

The conduct of the present study has received approval from the Scientific Council of the General Hospital 'Papageorgiou' (Protocol Number: 300, Date: 01/08/2018).

Given that participation in the study involves access to personal data, it should be noted that the European Union Directive on data confidentiality (95/46/EC) is strictly observed and implemented in Greece under Law 4624/2019 (Government Gazette A’ 137/29.08.2019). This directive stipulates that data usage is exclusively for research purposes.

Study criteria

The study included patients with stage 4-5 CKD who were being monitored at the outpatient clinics of six nephrology units in Greece (University General Hospital of Alexandroupoli, General Hospital of Thessaloniki “Papageorgiou”, General Hospital of Thessaloniki “Papanikolaou”, General Hospital of Xanthi, General Hospital of Edessa, General Hospital of Kavala). Individuals who agreed to participate were provided with informed consent forms, which detailed the research process and included terms of confidentiality.

Procedure

Participants were informed that they would need to complete three questionnaires before the educational intervention, attend the educational sessions, and then complete the same questionnaires again after a minimum of six months. In contrast, participants in the control group were only required to complete the questionnaires again after the same period. The first educational session included a 60-minute presentation covering the content detailed below. The second session consisted of a video screening followed by a discussion, with a total duration of 60 minutes. On average, the waiting period was 13 months for the intervention group and 14 months for the control group. The research process was conducted from October 2018 to December 2023.

The educational intervention was conducted in the form of a presentation using Microsoft PowerPoint (Microsoft Corporation, Redmond, WA, US) accompanied by the screening of a relevant video. The primary objective of the program was to provide comprehensive information regarding disease management through hemodialysis, peritoneal dialysis, and kidney transplantation. Specifically, the educational intervention was meticulously structured to cover various critical aspects of CKD management, including hemodialysis, peritoneal dialysis, and kidney transplantation. The presentation provided a detailed explanation of how hemodialysis mimics kidney function by filtering waste and excess fluids from the blood. It thoroughly described the hemodialysis process, emphasizing the simultaneous roles of diffusion, filtration, and osmosis in waste removal, fluid balance, and electrolyte restoration. Furthermore, patients were informed about the hemodialysis procedure, including how blood is drawn from the body, filtered through the hemodialysis machine, and returned to the body. The hemodialysis machine, its components, and its operation were presented in detail. Additionally, significant emphasis was placed on the types of vascular access used in hemodialysis, particularly arteriovenous fistulas (AVFs), arteriovenous grafts (AVGs), and central venous catheters. The training included information on how these access points are created, maintained, and monitored for complications such as infection or thrombosis. Furthermore, the intervention provided information on potential complications associated with hemodialysis (such as hypotension and muscle cramps).

Similarly, regarding peritoneal dialysis, patients were informed about the role of the peritoneal membrane as a natural filter during the peritoneal dialysis process, including the placement of the peritoneal catheter. The intervention covered both continuous ambulatory peritoneal dialysis (CAPD) and automated peritoneal dialysis (APD). A significant portion of the session was dedicated to teaching patients or their caregivers how to perform peritoneal dialysis independently. This included proper techniques for fluid exchanges, maintaining a clean environment using disinfectants to prevent infections, and recognizing signs of peritonitis or other complications. Patients were informed about the necessary lifestyle adjustments, such as dietary restrictions and fluid management, to complement peritoneal dialysis therapy. The intervention also emphasized the importance of adhering to prescribed treatment regimens and attending regular medical check-ups.

Moreover, the intervention included education on the pre-transplant evaluation process, including the required tests for selecting a suitable kidney transplant recipient. Detailed information was provided through the presentation about the surgical procedure, potential risks, and expected outcomes of kidney transplantation. Additionally, patients were informed about the required post-transplant care, including immunosuppressive therapy, monitoring for signs of organ rejection, and managing the increased risk of infections. The importance of adhering to medication regimens and attending regular medical follow-ups was emphasized.

Finally, information was provided about the challenges patients face on psychological, familial, social, and professional levels, along with suggestions for improving their mental health. The educational intervention utilized a patient-centered pedagogical approach, leveraging principles of adult education to enhance engagement and retention. The educational sessions were interactive, combining didactic teaching with presentations and personalized guidance. This approach ensures that the information is accessible and comprehensible, regardless of patients' prior knowledge or learning style. Following the presentation, patients watched an educational video available on the YouTube platform [20], which presents information on both hemodialysis and peritoneal dialysis in an interactive format. The hemodialysis video illustrates the process of hemodialysis, where blood is filtered outside the body using a machine to remove waste products and excess fluids. It covers the setup, procedure, and what patients can expect during treatment. The peritoneal dialysis video explains peritoneal dialysis, a method that uses the peritoneum as a natural filter to clear waste from the blood. It details the procedure, types of peritoneal dialysis, and patient considerations.

Tools and assessment

Quality of life was assessed using the Short Form of the Health Survey Questionnaire (SF-36) by Ware and colleagues [21]. The responses are given on two-point, three-point, and five-point Likert-type scales, ranging from 1 = "Yes" to 2 = "No," from 1 = "Very much" to 3 = "Not at all," and from 1 = "Not at all" to 5 = "Very much." The total score is expressed on a scale ranging from 0 to 100 points, with higher scores indicating a better quality of life. Mental resilience was evaluated using the Connor-Davidson Resilience Scale (CD-RISC) [22]. The responses are given on a five-point Likert-type scale, ranging from 0 = "Not at all true" to 4 = "Almost always true." The total score is expressed on a scale from 0 to 100, with higher scores indicating greater resilience. To assess psychiatric conditions, such as anxiety and depression, which often coexist with chronic illnesses, the Hospital Anxiety and Depression Scale (HADS) by Zigmond and Snaith was utilized [23]. This is an instrument consisting of 14 statements, of which 7 refer to depressive symptoms and the other 7 to anxiety symptoms. Responses are given on a 4-point Likert-type scale, ranging from 0 to 3. The total score is expressed on a scale from 0 to 21, with scores between 0 and 7 indicating the absence of anxiety or depression, scores between 8 and 10 indicating moderate levels of anxiety or depression, and scores above 11 reflecting high levels of anxiety or depression. CD-RISC and HADS use sum scores, while SF-36 uses a mean-based transformation for the final scores. All questionnaires have been validated on the Greek population [24-26]. Finally, to evaluate participants' satisfaction with the intervention, a satisfaction questionnaire specific to the intervention was administered (Appendix 1). This questionnaire consisted of nine statements where each participant indicated their level of agreement, with zero corresponding to "Not at all" and four to "Very much". The scale's total score ranged from 0 to 36. In this sample, the minimum score was 19, and the maximum score was 36. The mean score was 31, indicating that the majority of participants were quite satisfied with the intervention.

Statistical analysis

Data analyses were performed using IBM SPSS Statistics for Windows, Version 29.0. (Released 2022; IBM Corp., Armonk, New York, United States). The normality of the sample was assessed using the Shapiro-Wilk test due to the sample size (less than 50). The primary statistical analyses selected were non-parametric tests: the Wilcoxon signed-rank test to evaluate the intervention's effectiveness, the Mann-Whitney test to compare the control group with the intervention group, and Spearman’s correlation coefficient for assessing correlations. These statistical methods were deemed more appropriate than their parametric counterparts due to the absence of normal distribution in multiple levels of the variables, as identified by the Shapiro-Wilk test. Significance levels were two-tailed, and the statistical significance threshold was set at 0.05.

## Results

A total of 61 patients agreed to participate in the study. Participants were divided into two groups: the control group, consisting of 14 patients, and the intervention group, consisting of 47 patients. However, 14 patients from the intervention group (87.5%) and 2 from the control group (12.5%) were not included in the final analysis due to the death of 12 patients and the loss of follow-up for the remaining 4. As a result, the control group included 12 participants, and the intervention group included 33 participants. All participants demonstrated good cognitive functioning, enabling them to participate in the study. The demographic characteristics of the two groups are presented in Table 1.

**Table 1:** Demographic characteristics of patients

		Intervention Group	Control Group
		Ν (%)	Ν (%)
Gender	Men	22 (67%)	7 (58%)
	Women	11 (33%)	5 (42%)
Age Group	30-59	6 (18%)	2 (17%)
	60-69	8 (24%)	3 (25%)
	Over 70	19 (58%)	7 (58%)
Profession	Public employees	1 (3%)	0 (0%)
	Private employees	4 (12%)	0 (3%)
	Freelancer	1 (3%)	1 (8%)
	Retirees	24 (73%)	9 (75%)
	Unemployed	2 (6%)	2 (17%)
	Other	1 (3%)	0 (0%)
Education	Primary school	19 (58%)	9 (75%)
	Secondary education	7 (21%)	2 (17%)
	High school	7 (21%)	1 (8%)
Marital Status	Married	32 (97%)	11 (92%)
	Single	1 (3%)	1 (8%)
Insurance	Public	30 (88,2%)	12 (100%)
	Private	1 (2,9%)	0 (0%)
	Uninsured	2 (8,8%)	0 (0%)
Hospital	Alexandroupolis	7 (21%)	6 (50%)
	Papageorgiou	6 (18%)	4 (33%)
	Papanicolaou	12 (36%)	0 (0%)
	Xanthi	6 (18%)	0 (0%)
	Edessa	2 (6%)	0 (0%)
	Kavala	0 (0%)	2 (17%)

The satisfaction questionnaire consisted of nine statements where each participant indicated their level of agreement, with 0 corresponding to "Not at all" and 4 to "Very much". The scale's total score ranged from 0 to 36. In this sample, the minimum score was 19, and the maximum score was 36. The mean score was 31, indicating that the majority of participants were quite satisfied with the intervention.

Table 2 presents the changes in the mean scores of various parameters evaluated in the two patient groups. Overall, no significant differences were observed between the two groups in any parameter except for mental resilience and depression, which showed improvement in the control group compared to the intervention group.

**Table 2:** Changes in the median scores of various parameters evaluated in the two patient groups The Mann-Whitney U test was used for the comparison between the control group and the intervention group.

	Range	Intervention group	Control group	U-value	p
Mental Resilience					0.009
1st measurement	0-100	100	78.5	98.5	
2nd measurement	0-100	98	89.5		
Depression					0.009
1st measurement	0-21	5	9	98	
2nd measurement	0-21	5	6.5		
Anxiety					0.81
1st measurement	0-21	4	6	188.5	
2nd measurement	0-21	3	6.5		
Physical Functionality					0.62
1st measurement	0-100	60	47.5	178.5	
2nd measurement	0-100	75	57.5		
Physical Role					0.28
1st measurement	0-100	75	0	155	
2nd measurement	0-100	75	37.5		
Emotional Role					0.09
1st measurement	0-100	100	50	132.5	
2nd measurement	0-100	100	100		
Vitality					0.10
1st measurement	0-100	55	47.5	134	
2nd measurement	0-100	50	60		
Mental Functioning					0.37
1st measurement	0-100	68	60	162.5	
2nd measurement	0-100	60	68		
Social Functionality					0.15
1st measurement	0-100	75	68.7	141.5	
2nd measurement	0-100	75	87.5		
Physical Pain					0.19
1st measurement	0-100	77.5	77.5	146	
2nd measurement	0-100	77.5	95		
General Health					0.13
1st measurement	0-100	50	37.5	138.5	
2nd measurement	0-100	45	50		

Correlation between satisfaction with the intervention and the change in the scores of various parameters

A statistically significant negative correlation was found between the satisfaction scale and the parameters of anxiety and depression. Conversely, a significant positive correlation was observed with the social functioning score. Higher satisfaction with the intervention was associated with a significant reduction in anxiety and depression scores, as well as a significant increase in social functioning scores. Additionally, a marginally significant positive correlation was noted between the satisfaction scale and mental resilience (Table 3).

**Table 3:** Correlation between satisfaction with the intervention and the change in the scores of various parameters

	r	p
Mental Resilience	0.32	0.07
Depression	-0.44	0.01
Anxiety	-0.38	0.03
Physical Functioning	0.20	0.25
Physical Role	0.14	0.44
Emotional Role	0.02	0.91
Vitality	0.08	0.65
Mental Functioning	0.15	0.41
Social Functioning	0.40	0.02
Physical Pain	0.09	0.61
General Health	0.13	0.47

## Discussion

The purpose of this study was to examine the impact of an educational intervention aimed at improving patient knowledge about kidney disease and its treatment options on the quality of life, depression, anxiety, and psychological resilience of patients with stage 4-5 CKD. According to the available literature, this study is the first to examine such an intervention's overall effects on these variables, particularly in the Greek context. The study assessed the intervention's effectiveness in increasing psychological resilience, reducing depression and anxiety levels, and improving quality of life. Additionally, for the first time, the level of satisfaction with the intervention and its correlation with all study parameters were evaluated.

The findings indicated that no significant differences were observed between the two groups in terms of anxiety and all aspects of quality of life. However, regarding psychological resilience and depression, a significant improvement was noted in the control group compared to the intervention group. The reasons for this unexpected result cannot be identified within the scope of the present study. This improvement may have been influenced by variables not measurable within the study such as personal events in the patients' lives or broader societal conditions during the study period.

Similarly, the outcomes of a recent study on the efficacy of an educational intervention in reducing depression and anxiety showed no significant differences in anxiety scores [27]. Other meta-analyses concluded that educational interventions for patients with chronic kidney disease are moderately effective in reducing depression levels and weakly effective in improving quality of life and anxiety in this population [14,28]. These studies, however, included individuals with earlier stages of chronic kidney failure, unlike the present study, which exclusively included patients at stages 4-5 CKD.

One potential reason for the non-statistically significant findings in this study could be the extended time gap between the initial parameter measurements, the intervention, and the follow-up measurements for some participants. Specifically, the research process began in October 2018 and extended until December 2023. This prolonged research period was due to the COVID-19 pandemic, which emerged shortly after the study's initiation and inevitably disrupted data collection. For some participants, the intervention occurred several years after the first measurement or long before the second measurement, introducing the risk of maturation bias, referring to changes in participants over time that are unrelated to the intervention. Over five years, participants may have experienced changes due to aging, life experiences, or other processes. Such changes could influence the study’s measured outcomes, with maturation effects being mistaken for intervention effects or even overshadowing the intervention's actual impact.

Additionally, since this time frame coincided with the COVID-19 pandemic, the recorded levels of psychological resilience, quality of life, anxiety, and depression might have been influenced by pandemic-related conditions. It is well-documented that lockdowns during the pandemic were associated with increased levels of depression and anxiety, as well as decreased quality of life, particularly in patients with CKD [29,30]. These external factors could confound the results, making it difficult to attribute changes (or the absence of changes) solely to the intervention under study. Another potential reason for the lack of significant findings in this study might be the relatively small sample size, particularly in the control group.

Based on these results, one might argue that interventions of this type do not offer substantial benefits. However, the study demonstrated that satisfaction with the intervention was significantly correlated with reductions in anxiety and depression, as well as improvements in patients’ social functioning. Furthermore, there was a marginal improvement in the psychological resilience of participants in the educational program. This notable finding suggests that participants who reported greater satisfaction with the intervention experienced significant reductions in anxiety and depression, enhanced social functioning, and slight improvement in their psychological resilience. Therefore, the level of satisfaction with the intervention emerges as a critical factor determining its effectiveness. Efforts to improve educational interventions are of paramount importance. Tailoring the intervention to align with the individual characteristics of each patient may also enhance its effectiveness, ensuring that it addresses the unique needs of each participant.

The advent of the COVID-19 pandemic during the study constitutes a major limitation, as it led to a time extension and clearly affected the participants' psychology in various ways (confinement, insecurity, etc.). It is essential to note that the intervention in question was designed for informational purposes and did not follow any established models of educational interventions, such as the Patient Therapeutic Education Guide [10]. Additionally, it was not informed by any psychoeducational or psychocommunicative framework and lacked direction from a targeted psychotherapeutic approach such as Cognitive Behavioral Therapy (CBT). This indicates room for improvement, and future studies should utilize targeted frameworks to evaluate the impact of education on psychological resilience, anxiety, depression, and quality of life in Greek populations with stage four and above renal failure.

Finally, this study did not account for other potential confounding variables that could influence the outcomes such as personality traits, the patient’s support network, intolerance of uncertainty, the time since diagnosis, or the duration of treatment. Future research should incorporate these variables using advanced statistical techniques, such as moderation and mediation analyses, to better understand their role in shaping outcomes.

## Conclusions

In conclusion, despite the unexpected outcomes of this study, which should be interpreted in light of the COVID-19 pandemic during which the research was conducted, the reduction in anxiety and depression, improvement in social functioning, and marginal enhancement of psychological resilience among patients who reported high satisfaction with the intervention underscore the significant value of comprehensive patient education explored in this study. Future research should include a larger sample size and consider additional variables (e.g., personality traits and support networks) or alternative methodological approaches such as collecting and analyzing qualitative data or designing interventions grounded in theoretical frameworks. Most importantly, educational interventions should be refined to address the specific needs of each patient better. This study represents the first step in this direction within the Greek context and provides a foundation for further research aimed at advancing the field and improving psychosomatic care for a population that, due to its multiple morbidities, has an acute need for such support. These findings highlight the importance and necessity of educating patients on matters related to their disease and treatment.

## Appendices

Intervention satisfaction questionnaire

Instruction:

Please rate the following statements based on your experience with the intervention, using the scale below:

0 = Not at all

1 = A little

2 = Moderate

3 = Very much

4 = Extremely

1.       I am satisfied with the overall intervention/information provided.

2.       I am satisfied with the duration of the intervention.

3.       I am satisfied with the content presented during the intervention.

4.       I am satisfied with the tools used (e.g., PowerPoint presentation, videos) during the intervention.

5.       I am satisfied with the healthcare professional who conducted the intervention.

6.       I believe this intervention will help me choose the appropriate treatment for my condition.

7.       I believe this intervention will help me improve my quality of life.

8.       I believe this intervention will help me reduce negative thoughts related to my condition.

9.       I believe this intervention will help me reduce the anxiety caused by my condition.
